# Size and location of intracranial hemorrhage in acute stroke presentations: correlation with baseline NIHSS and relevance to direct-to-angiography workflows

**DOI:** 10.3389/fneur.2026.1712607

**Published:** 2026-09-08

**Authors:** Anh Nguyen, Mira Katan, Raphael Guzman, Jehuda Soleman, Christian Nickel, Marios-Nikos Psychogios, Alex Brehm

**Affiliations:** 1 Department of Diagnostic and Interventional Neuroradiology, University Hospital Base, Basel, Switzerland; 2 University Basel, Basel, Switzerland; 3 Department of Neurology, University Hospital Basel, Basel, Switzerland; 4 Department of Pediatric Neurosurgery, University Children’s Hospital Basel (UKB), Basel, Switzerland; 5 Department of Emergency Medicine, University Hospital Basel, Basel, Switzerland

**Keywords:** computed tomography, direct-to-angiography, epidemiology, intracranial hemorrhage, stroke, stroke severity (NIHSS)

## Abstract

**Background and purpose:**

Direct-to-angiography (DTA) workflows for suspected large vessel occlusion stroke reduce treatment delays by bypassing emergency-department imaging, but their safety depends on excluding intracranial hemorrhage (ICH) on flat-panel detector CT (FDCT), where posterior fossa visualization is limited by beam-hardening artifacts. Epidemiological data on ICH size and location relative to stroke severity are limited. We characterized ICH type, volume, and location in acute stroke presentations and their relationship with baseline NIHSS.

**Materials and methods:**

We retrospectively analyzed 394 patients with imaging-confirmed spontaneous ICH from the prospective Swiss Stroke Registry at a high-volume comprehensive stroke center. Hemorrhages were classified by type, location, and volume using semi-automated segmentation. Associations with baseline NIHSS were assessed using Spearman’s correlation, Wilcoxon rank-sum tests (NIHSS <7 vs. ≥7), and generalized additive and ordinal regression models adjusted for age, location, and secondary extension.

**Results:**

Intraparenchymal hemorrhage accounted for 89% of cases, followed by subarachnoid (7%), intraventricular (3%), and subdural (<1%). Median volume was 18 mL (IQR 5–51). Volume correlated with NIHSS (Spearman *ρ* = 0.56, 95% CI 0.48–0.64, *p* < 0.001), with a significant nonlinear association in adjusted models. Patients with NIHSS ≥7 had larger volumes than those with NIHSS <7 (median 38 vs. 6 mL, *p* < 0.001). Lobar hemorrhages had the largest volumes. In contrast, infratentorial hemorrhages, particularly brainstem bleeds, produced severe deficits at small volumes (median 6 mL) and were not associated with larger volumes after adjustment, indicating that location influences severity independently of size. Isolated infratentorial hemorrhage accounted for only 2.8% of NIHSS ≥7 patients.

**Conclusion:**

Severity in supratentorial ICH is volume-dependent, whereas location, particularly brainstem involvement, drives severity in isolated infratentorial ICH independently of volume. Although rare among high-NIHSS patients, these small-volume bleeds may be hardest to detect on FDCT and warrant attention to posterior fossa image quality in DTA workflows. Because detectability was not measured, prospective paired FDCT-MDCT studies are needed.

## 1 Introduction

Stroke remains a leading global cause of disability and mortality, with outcomes strongly dependent on timely and accurate diagnosis (1–3). Acute strokes are broadly divided into ischemic, caused by arterial occlusion, and hemorrhagic, caused by spontaneous intracranial hemorrhage, each requiring fundamentally different treatment strategies. In ischemic stroke, the principle of “time is brain” underscores the urgency of reperfusion therapy, with pooled randomized trial data showing that earlier treatment initiation directly improves outcomes (2, 3).

Conventional stroke workflows involve initial neurological assessment, non-contrast multidetector CT (MDCT) to exclude ICH, followed by intravenous thrombolysis (IVT) when indicated, and subsequent MDCT angiography ± perfusion to identify large vessel occlusion (LVO) prior to endovascular therapy. While effective, this multistep pathway typically introduces delays of around 60 min from hospital arrival to treatment initiation (4). Direct-to-angiography (DTA) approaches have therefore been proposed to accelerate care. In these workflows, patients with suspected LVO, often defined by a National Institutes of Health Stroke Scale (NIHSS) score ≥7 due to higher LVO probability (5), are transferred directly to the angiography suite, where flat-panel detector CT (FDCT) excludes hemorrhage before proceeding to thrombectomy. Observational studies and early randomized trials suggest that DTA can reduce door-to-treatment time by more than 30 min, with potential improvements in outcomes (6–17).

A critical safety requirement of DTA is reliable exclusion of intracranial hemorrhage prior to IVT. Approximately 20% of patients with NIHSS ≥7 have hemorrhagic rather than ischemic stroke, and FDCT image quality in the posterior fossa remains a concern due to beam-hardening artifacts (18–20). While FDCT has shown high accuracy for supratentorial bleeds, small-volume or infratentorial hemorrhages may be more difficult to detect. Understanding the epidemiology of intracranial hemorrhage in acute stroke presentations, specifically the frequency, size, and location of hemorrhages in relation to NIHSS, is therefore essential to estimate how often such challenging cases may arise in the DTA context.

The present study aimed to address this gap by systematically characterizing acute intracranial hemorrhage in a large cohort of patients presenting with stroke symptoms. Our objectives were threefold: (1) to describe the frequency, size, and anatomical location of hemorrhages; (2) to examine the relationship between hemorrhage volume and clinical severity as measured by NIHSS; and (3) to evaluate the contribution of hemorrhage location to stroke severity, with particular focus on infratentorial bleeds that may pose challenges for FDCT-based DTA workflows.

## 2 Methods

### 2.1 Design and setting

We conducted a retrospective, single-center analysis using data from the Swiss Stroke Registry, a comprehensive national stroke database that enrolls patients admitted to certified stroke units with acute ischemic or hemorrhagic stroke. The study site is a high-volume comprehensive stroke center (University Hospital Basel) providing >99% coverage of regional stroke alerts (“mothership” model).

### 2.2 Patient selection

Adults aged ≥18 years admitted between 01.01.2020 and 31.12.2022 with spontaneous intracranial hemorrhage confirmed on MDCT or FDCT were included. We excluded cases of traumatic hemorrhage (including epidural hematoma) and recurrent intracranial hemorrhage within 3 months after initial hemorrhagic event. Cases in which MRI was the sole diagnostic imaging modality for intracranial hemorrhage were excluded, because MRI-derived volumetric measurements are not directly comparable to CT-based semi-automated segmentation due to differences in signal characteristics and susceptibility-weighted sequence blooming artifacts (18, 19). An exception was made for cases in which non-contrast MDCT or FDCT was subsequently performed within 6 h of the initial MRI, provided that the radiology report documented hemorrhage stability, defined as no significant change in hemorrhage size and no new hemorrhage on follow-up CT. In these cases, CT-derived measurements were used for volumetric analysis.

### 2.3 Ethics

The institutional review board (Ethikkommission Nordwest- und Zentralschweiz, project ID 2023-02219) approved the study with a waiver of informed consent.

### 2.4 Clinical data collection

Demographic and clinical data were extracted from the Swiss Stroke Registry and supplemented by electronic health records where values were missing. Variables collected included age, sex, vascular risk factors (hypertension, diabetes, prior stroke, atrial fibrillation, smoking status, antithrombotic therapy), and clinical presentation data (NIHSS, Glasgow Coma Scale, systolic blood pressure, symptom onset time). NIHSS was assessed at admission by an attending stroke neurologist.

### 2.5 Imaging acquisition and review

At presentation, all patients underwent neuroimaging to confirm the presence, type, and extent of hemorrhage. Non-contrast MDCT and/or FDCT was used according to clinical workflow. MDCT scans were acquired on SOMATOM Definition AS+ and SOMATOM Force scanners (Siemens Healthineers). FDCT was performed on an Axiom Artis system (Siemens Healthineers). Hemorrhage characteristics, including compartmental extension, were assessed on this initial imaging only; follow-up imaging and hematoma expansion were not evaluated in this study.

Hemorrhage volume was quantified using a semi-automated segmentation tool (Sectra Volume Measurement Tool) by a single trained rater. Given that reliable delineation of blood volume within the subarachnoid space is not feasible with this method, isolated SAH volume was not quantified or included in volumetric analyses. Intraventricular hemorrhage (IVH) severity was graded using the Intraventricular Hemorrhage Score (21), and aneurysmal subarachnoid hemorrhage (SAH) severity using the modified Fisher scale (22). An aneurysmal etiology was recorded when an underlying aneurysm was identified on CTA or DSA. The presumed etiology for all hemorrhage types was determined based on the final diagnosis documented in the discharge report following the complete diagnostic stroke workup, incorporating relevant imaging, laboratory, and clinical findings.

Hemorrhages were classified by type [intracerebral hemorrhage (ICH), SAH, IVH, subdural hematoma (SDH)] and by anatomical location (lobar, deep, brainstem, cerebellar, sulcal subarachnoid, cisternal subarachnoid, subdural). For ICH, the primary location was assigned according to the CHARTS criteria (21) based on the hemorrhage epicenter and categorized as lobar, deep, infratentorial, or uncertain, including probable lobar, probable deep, and holocephalic cases. In addition, all hemorrhages were grouped using a broader anatomical classification as isolated supratentorial, isolated infratentorial, or combined supra- and infratentorial.

Secondary extension into other intracranial compartments and mass effect were recorded for each hemorrhage type. Extension was recorded both categorically (present/absent, used in the multivariable model) and as a single pooled extension volume summed across all involved compartments, reflecting total additional hemorrhage burden rather than a compartment-specific measurement. Location assignment was performed blinded to NIHSS. All images were initially reviewed by a radiology resident and validated by a senior board-certified neuroradiologist. An exploratory slice-visibility analysis in infratentorial DTA-eligible patients is described in Supplementary material 1.

### 2.6 Statistical analysis

The primary endpoint was baseline NIHSS, analyzed both as continuous measure (for the volume association) and as an ordinal severity category. The primary analysis population was ICH patients with complete NIHSS (*n* = 333). No formal sample size calculation was performed. The available registry cohort defined the sample, and subgroup analyses are correspondingly underpowered and reported as exploratory. Statistical analyses were conducted using R (version 4.4.2). Continuous variables are reported as medians with interquartile ranges (IQR) and categorical variables as counts with percentages. Normality was assessed using frequency histograms, Q–Q plots, and the Shapiro–Wilk test. Patients with missing NIHSS values (*n* = 25) were excluded from analyses requiring NIHSS. Although the overall number of missing NIHSS values was low, missingness occurred predominantly in patients with reduced consciousness, as reflected by available GCS scores (no missing data), and was therefore considered unlikely to be completely at random. Complete-case analyses were interpreted cautiously, and the potential for underestimation of clinical severity among excluded patients was considered in the interpretation of results.

Analyses were structured at three levels. (1) descriptive analyses across all hemorrhage types (*n* = 394); (2) primary analyses were restricted to ICH patients with complete NIHSS data (*n* = 333), because ICH comprised 89% of the patient cohort and the volume-NIHSS relationship is likely to vary by hemorrhage subtype; and (3) a prespecified subgroup analysis in ICH patients with NIHSS ≥7 and symptom onset-to-arrival time ≤360 min (*n* = 163), representing a population typical of real-world DTA triage candidates (11).

The association between primary hemorrhage volume and NIHSS was assessed using Spearman’s rank correlation with bootstrap 95% confidence intervals. Hemorrhage volumes were compared between NIHSS <7 and ≥7 groups using the Wilcoxon rank-sum test with Cliff’s delta as the effect size measure. Associations between hemorrhage volume and NIHSS were assessed using Spearman’s rank correlation, reported overall and stratified by hemorrhage location. Adjustment variables were selected using a directed acyclic graph (DAG; Supplementary material 2) to support transparent model specification and avoid adjustment for mediators or colliders.

Nonlinear relationship between NIHSS and hemorrhage volume was further examined using generalized additive models (GAMs), adjusted for age, hemorrhage location (deep, lobar, infratentorial), and secondary extension (binary). Model adequacy was assessed using residual Q-Q-plots and the gam.check. The ordinal logistic regression modeled NIHSS severity category (mild <7, moderate 7–13, severe 14–21, very severe ≥22) with the same covariates plus extension type (none/IVH/SAH/combined IVH + SAH). The proportional odds assumption was evaluated using the Brant test, with violations handled by a partial proportional-odds model. All models used complete-case data. No correction for multiple comparisons was applied; results should be considered exploratory.

To improve model stability, less frequent hemorrhage types (SAH, SDH, IVH) were grouped into an “other” category, allowing comparisons across lobar, deep, infratentorial, and other hemorrhages. Interaction terms were excluded from the final model due to small subgroup sizes and lack of significant model improvement. Outside this three-level structure, a *post hoc* exploratory analysis was performed in the SAH subgroup (*n* = 30). The distribution of GCS was assessed for multimodality using Hartigan’s dip test. GCS and NIHSS were compared between aneurysmal and non-aneurysmal etiologies using the Wilcoxon rank-sum test.

## 3 Results

### 3.1 Study cohort and analysis populations

**Figure 1:**
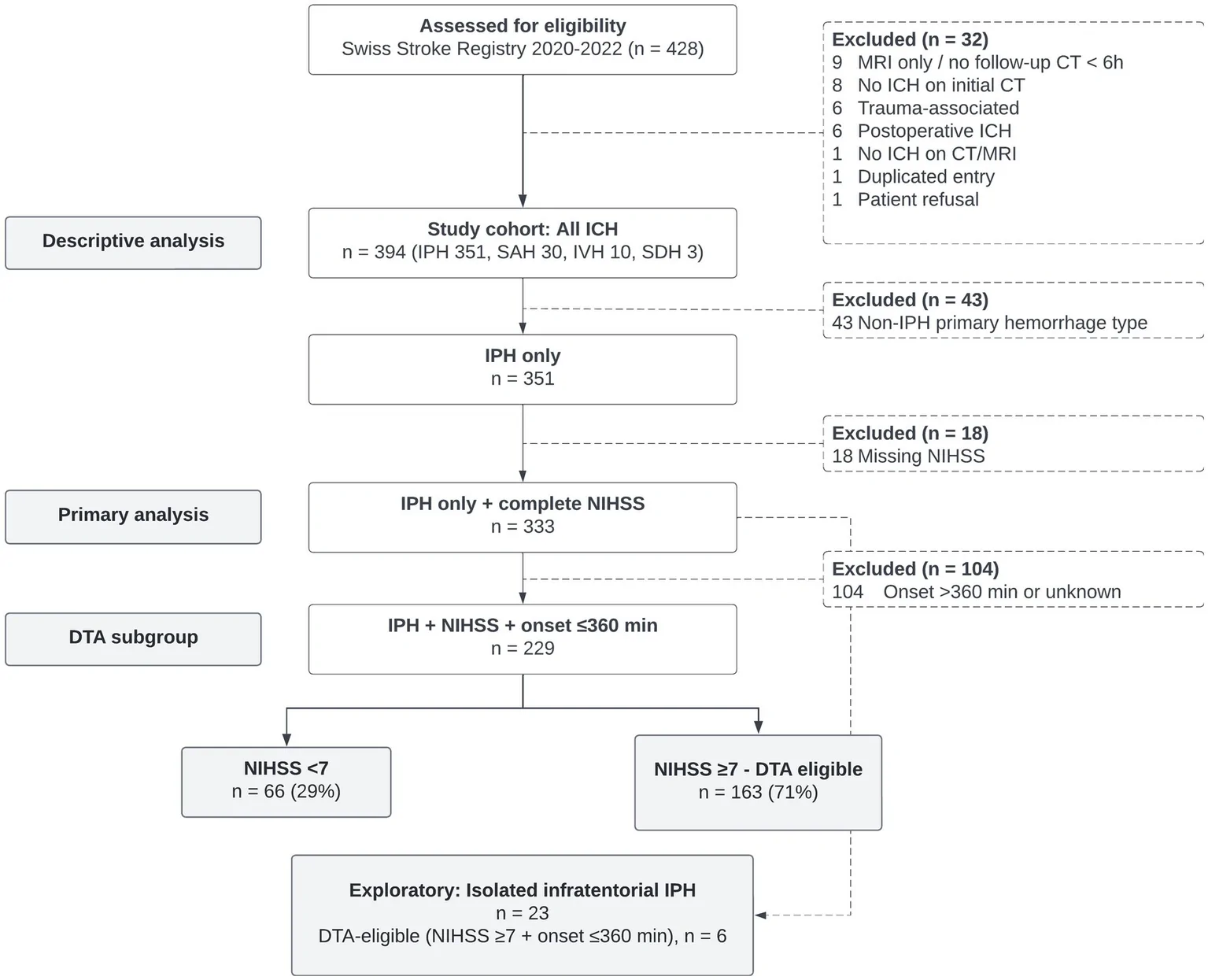
Flow diagram of patient selection. Flowchart of patient selection and analysis populations. Patients were identified from the Swiss Stroke Registry at the University Hospital Basel between January 2020 and December 2022. After exclusion of 32 patients, 394 with confirmed spontaneous intracranial hemorrhage formed the study cohort (descriptive analysis). Primary analyses were restricted to 333 patients with intracerebral hemorrhage and complete National Institutes of Health Stroke Scale (NIHSS) data. A prespecified subgroup analysis included 229 patients with additional known symptom onset-to-arrival time ≤360 min, of whom 163 (71%) had NIHSS ≥7 and met direct-to-angiography (DTA) eligibility criteria. An exploratory analysis was performed in 23 patients with isolated infratentorial ICH, of whom 6 met DTA eligibility criteria. ICH, intracerebral hemorrhage; SAH, subarachnoid hemorrhage; IVH, intraventricular hemorrhage; SDH, subdural hematoma; NIHSS, National Institutes of Health Stroke Scale; CT, computed tomography; MRI, magnetic resonance imaging; DTA, direct-to-angiography.

From an initial cohort of 415 patients, 394 met the inclusion criteria and formed the study cohort (Figure 1). The median age was 75 years (IQR, 62–83), and 196 patients (49%) were female (Table 1; Supplementary material 3). NIHSS was missing in 25 patients (6.3%). ICH was the predominant hemorrhage subtype, present in 351 patients (89%). Therefore, primary analyses were restricted ICH patients (*n* = 333) with complete NIHSS data. Among these, 163 met DTA eligibility criteria (symptom onset to arrival ≤360 min, NIHSS ≥7). Ten patients underwent initial imaging with FDCT as part of a direct-to-angiography pathway; their clinical and imaging characteristics are summarized in Supplementary material 4. Deviations from the DTA pathway in the remaining eligible patients were attributable to factors including presentation during on-call hours, transfer from an external hospital, or diagnostic uncertainty regarding symptom onset in patients with decreased level of consciousness.

**Table 1:** Patient and clinical characteristics.

	Total		ICH		IVH		SAH		SDH	
Count	394		351		10		30		3	
Patient demographics										
Age	75	(62, 83)	76	(63, 84)	66	(30, 85)*	65	(57, 76)	87	(71, 87)*
Gender, female	196	(50%)	170	(48%)	6	(60%)	20	(67%)	0	
Premorbid mRS	1	(0, 2)	1	(0, 2)	0	(0, 2)*	0	(0, 2)*	2	(2, 2)*
Medical history										
Previous ischemic stroke	68	(17%)	61	(17%)	1	(10%)	5	(17%)	1	(33%)
Previous intracranial hemorrhage	35	(9%)	32	(9%)	1	(10%)	2	(7%)	0	
Hypertension	298	(76%)	276	(79%)	6	(60%)	15	(50%)	1	(33%)
Diabetes mellitus	70	(18%)	64	(18%)	1	(10%)	4	(13%)	1	(33%)
Hypercholesterolemia	125	(32%)	112	(32%)	2	(20%)	11	(37%)	0	
Active smoking	66	(17%)	56	(16%)	1	(10%)	9	(30%)	0	
Atrial fibrillation	86	(22%)	78	(22%)	2	(20%)	4	(13%)	2	(67%)
Anticoagulant use	87	(22%)	82	(23%)	2	(20%)	2	(7%)	1	(33%)
Antiplatelet use	102	(26%)	92	(26%)	1	(10%)	8	(27%)	1	(33%)
Clinical presentation										
NIHSS	10	(3, 18)	11	(4, 19)	1	(0, 32)*	2	(0, 40)*	8	(3, 25)*
GCS	14	(10, 15)	14	(10, 15)	14	(3, 15)*	14	(12, 15)	14	(8, 14)*
Systolic blood pressure	160	(140, 183)	161	(140, 185)	157	(113, 220)*	155	(105, 230)*	166	(149, 182)*
Wake-up stroke	40	(10%)	37	(11%)	0		3	(10%)	0	
Transfer from referring hospital	29	(7%)	25	(7%)	2	(20%)	2	(7%)	0	
Time (onset-admission)	150	(66, 533)	159	(66, 553)	192	(63, 7,948)*	98	(37, 1,253)*	1,040	(1,040, 1,040)*

Values are median (IQR) or count (%). * Range reported instead of IQR for groups with *n* < 30. ICH, intracerebral hemorrhage; SAH, subarachnoid hemorrhage; IVH, intraventricular hemorrhage; SDH, subdural hematoma; mRS, modified Rankin Scale; NIHSS, National Institutes of Health Stroke Scale; GCS, Glasgow Coma Scale. IVH and SDH subgroups are shown for descriptive completeness; small sample sizes preclude formal comparison.

### 3.2 Hemorrhage type, location, volume, and etiology

ICH was the most frequent hemorrhage type, occurring in 351 of 394 patients (89%), followed by SAH (7%), isolated IVH (3%), and SDH (<1%) (Table 2; Supplementary material 5). Most hemorrhages were isolated supratentorial (68%), whereas isolated infratentorial hemorrhages (7%) and combined supra- and infratentorial involvement (25%) were less frequent. Secondary extension into additional compartments occurred in approximately half of patients, most commonly into the ventricles (36%) and subarachnoid space (28%). Mass effect was present in 38% and was most frequent among lobar hemorrhages.

**Table 2:** Bleeding characteristics.

	Total		ICH		IVH		SAH		SDH	
Count	394		351		10		30		3	
Volume										
Primary hemorrhage	15	(5, 42)	16	(5, 45)	9	(2, 15)	24	(2, 86)*	136	(70, 244)*
Secondary extension	0.3	(0, 6)	0.5	(0, 7)	0.4	(0, 3)	0	(0, 6)*	0	(0, 8)*
Total hemorrhage volume	19	(5, 51)	20	(5, 54)	9.5	(2, 19)	24	(2, 86)*	136	(70, 252)*
Hemorrhage location										
Supratentorial only	268	(68%)	251	(72%)	12	(40%)	3	(30%)	2	(67%)
Infratentorial only	24	(6%)	23	(7%)	1	(3%)	0		0	
Supra- and infratentorial	102	(26%)	77	(22%)	17	(57%)	7	(70%)	1	(33%)
Location of extension										
Intracerebral	25	(6%)	20	(6%)	4	(13%)	1	(10%)	0	
Subarachnoid	98	(25%)	95	(27%)	0		1	(10%)	2	(67%)
Intraventricular	162	(41%)	147	(42%)	15	(50%)	0		0	
Subdural	30	(8%)	30	(9%)	0		0		0	
Mass effect										
Midline shift (*n*, %)	151	(38%)	143	(41%)	3	(10%)	3	(30%)	2	(67%)
Midline shift (in mm)	0	(0, 5)	0	(0, 6)	0	(0, 0)	0	(0, 8)*	10	(0, 17)*
Herniation	112	(28%)	105	(30%)	5	(17%)	1	(10%)	1	(33%)
Hydrocephalus	114	(29%)	94	(27%)	12	(40%)	7	(70%)	1	(33%)

Values are median (IQR) or count (%). *Range reported instead of IQR for groups with *n* < 30. ICH, intracerebral hemorrhage; SAH, subarachnoid hemorrhage; IVH, intraventricular hemorrhage; SDH, subdural hematoma. IVH and SDH subgroups are shown for descriptive completeness; small sample sizes preclude formal comparison.

Hemorrhage volumes varied substantially across hemorrhage types and locations. Overall median hemorrhage volume was 18 mL (IQR, 5–51), with the largest median volume observed among lobar hemorrhages. Infratentorial hemorrhage volumes were generally smaller, with a median of 10 mL, respectively. Hypertension was the most common presumed etiology (38%), particularly in deep and cerebellar hemorrhages, while cerebral amyloid angiopathy was the most frequent presumed etiology among lobar hemorrhages (75%).

### 3.3 Clinical severity in the SAH subgroup

Among the 30 patients with SAH, GCS scores clustered at 3–5 and 13–15 with few intermediate values, consistent with a bimodal distribution (dip test D = 0.117, *p* = 0.001), whereas NIHSS scores were predominantly low and right skewed. GCS was significantly lower in aneurysmal (*n* = 16) than non-aneurysmal SAH (*n* = 14, median 13 (IQR 4.5–14) vs. 15 (IQR 15–15), *p* < 0.001), and all five patients with GCS ≤ 8 had an underlying aneurysm. NIHSS showed a similar but non-significant trend (median 7 vs. 2, *p* = 0.055).

### 3.4 Association between ICH volume and NIHSS

**Figure 2:**
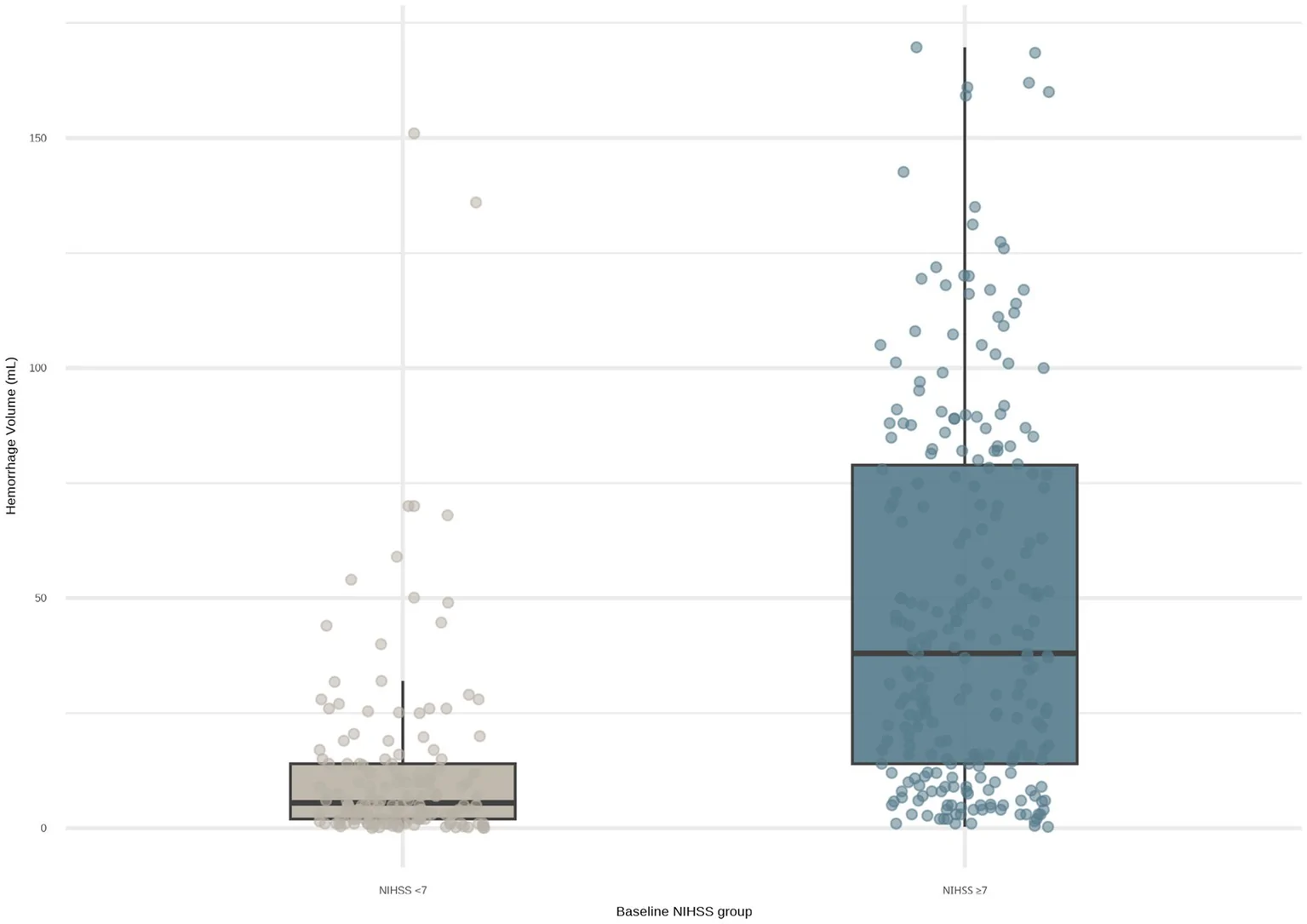
Distribution of hemorrhage volume by baseline NIHSS category. Patients with NIHSS ≥7 had significantly larger hemorrhage volumes compared with those with NIHSS <7 (median 38 mL vs. 6 mL, Wilcoxon rank-sum *p* < 0.001). The NIHSS ≥7 group also showed a broader range of values with more extreme outliers. NIHSS, National Institutes of Health Stroke Scale.

Among ICH patients with complete NIHSS data (*n* = 333), primary hemorrhage volume was moderately associated with higher NIHSS (Spearman *ρ* = 0.562, 95% CI 0.477–0.640, *p* < 0.001; Figure 2). Location-stratified Spearman correlations are reported in Supplementary material 6. Patients with NIHSS ≥7 had significantly larger hemorrhage volumes than those with NIHSS <7 (median, 38 mL (IQR, 14–79) vs. 6 mL (IQR, 2–14); *p* < 0.001), corresponding to a median difference of 32 mL (95% CI, 25–39). Secondary extension was more common in the NIHSS ≥7 group (43% vs. 6%), particularly into ventricular (54% vs. 16%) and subarachnoid (30% vs. 15%) spaces (Supplementary material 7).

**Figure 3:**
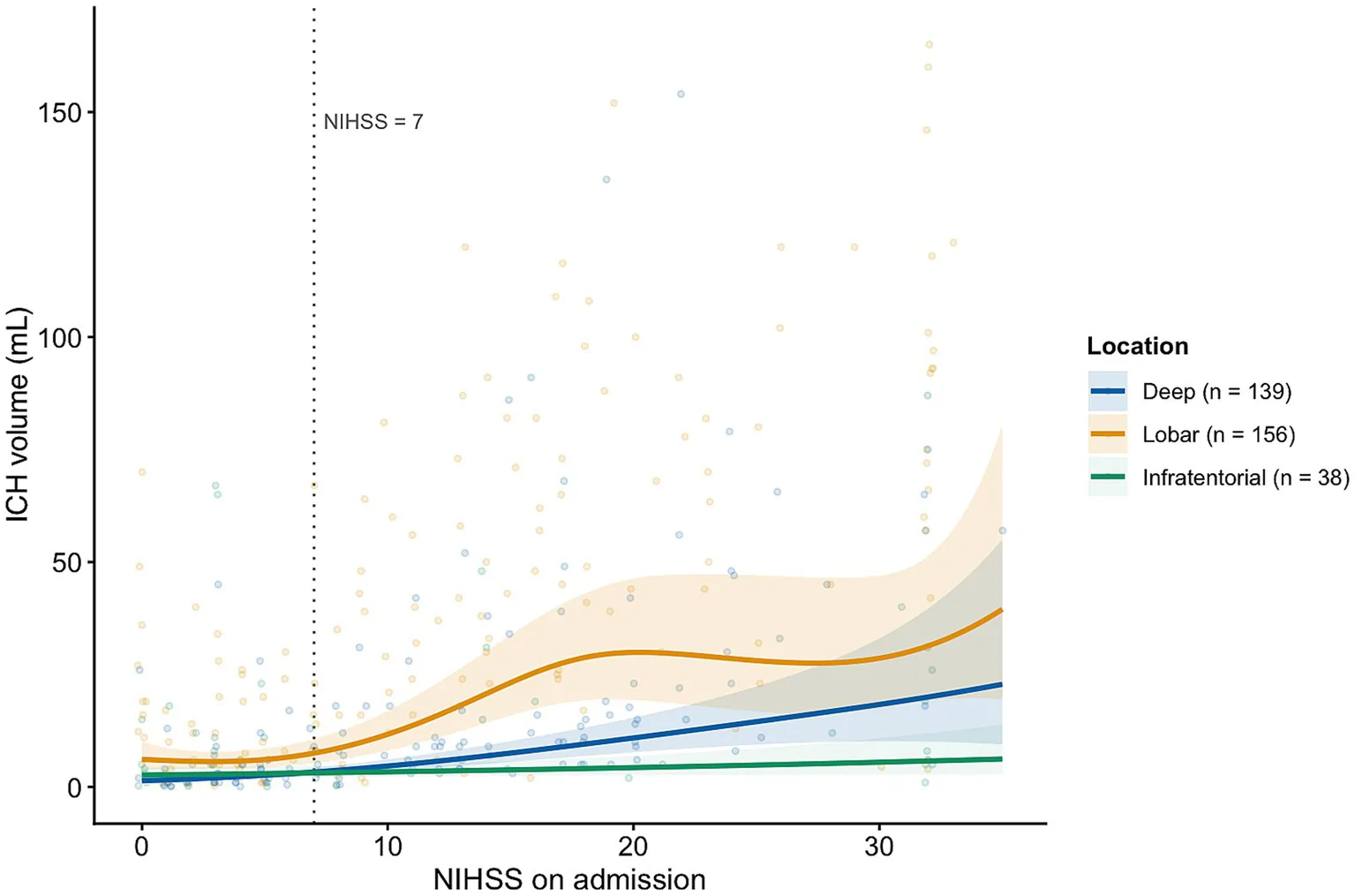
Location-stratified association between baseline *NIHSS* and intracerebral hemorrhage volume. Curves and shaded 95% confidence intervals represent location-specific GAM smooths, fitted on log-transformed IH volume and adjusted for age and hemorrhage extension into intraventricular and/or subarachnoid space, shown on the original scale (*n* = 333). The infratentorial curve is approximately linear and should be interpreted with caution given the small sample size (*n* = 38). The dotted vertical line marks *NIHSS* = 7, the institutional threshold for direct-to-angiography triage. Curves are shown up to *NIHSS* = 35; three patients with higher scores were included in the model but lie beyond the displayed range. NIHSS, National Institutes of Health Stroke Scale; GAM, generalized additive model.

In the GAM with log-transformed primary hemorrhage volume as outcome, NIHSS demonstrated a significant nonlinear association with hemorrhage volume, explaining 50.5% of the deviance (adjusted *R*^2^ = 0.49; smooth term: (EDF = 3.57, *F* = 21.73); *p* < 0.001) (Figure 3). The unadjusted association between mean hemorrhage volume by NIHSS score intervals is shown in Supplementary material 8. Compared to deep hemorrhages, lobar hemorrhages were associated with significantly larger volumes (*β* = 0.97, *p* < 0.001), corresponding to a 2.64-fold increase on a natural scale, while infratentorial location was not significantly associated with volume after adjustment (*β* = −0.14; *p* = 0.52). Secondary extension was associated with a 2.46-fold increased hemorrhage volume (*β* = 0.90, *p* < 0.001), while age was not significantly associated with volume.

### 3.5 Volume-adjusted association of ICH location and secondary extension with NIHSS severity

In ordinal logistic regression, primary hemorrhage volume showed the strongest association with higher NIHSS severity (OR per log-unit 2.50, 95% CI 2.04–3.11, *p* < 0.001). IVH extension was associated with higher NIHSS severity (OR 3.04, 95% CI 1.76–5.28, *p* < 0.001), whereas isolated SAH extension was not (OR 0.86, 95% CI 0.43–1.69, *p* = 0.63). Compared to deep ICH, lobar ICH was associated with lower NIHSS severity (OR 0.58, 95% CI 0.34–0.97, *p* = 0.039), and age was also independently associated with severity (OR per year 1.02, 95% CI 1.003–1.032, *p* = 0.026). The proportional-odds assumption was violated for infratentorial location (Brant test χ^2^ = 35.91, *p* < 0.001), and the proportional-odds model showed infratentorial hemorrhages more common among mild presentations (20% of NIHSS <7 vs. 7% of NIHSS ≥7).

### 3.6 Isolated infratentorial hemorrhages and DTA-eligible subgroup

**Figure 4:**
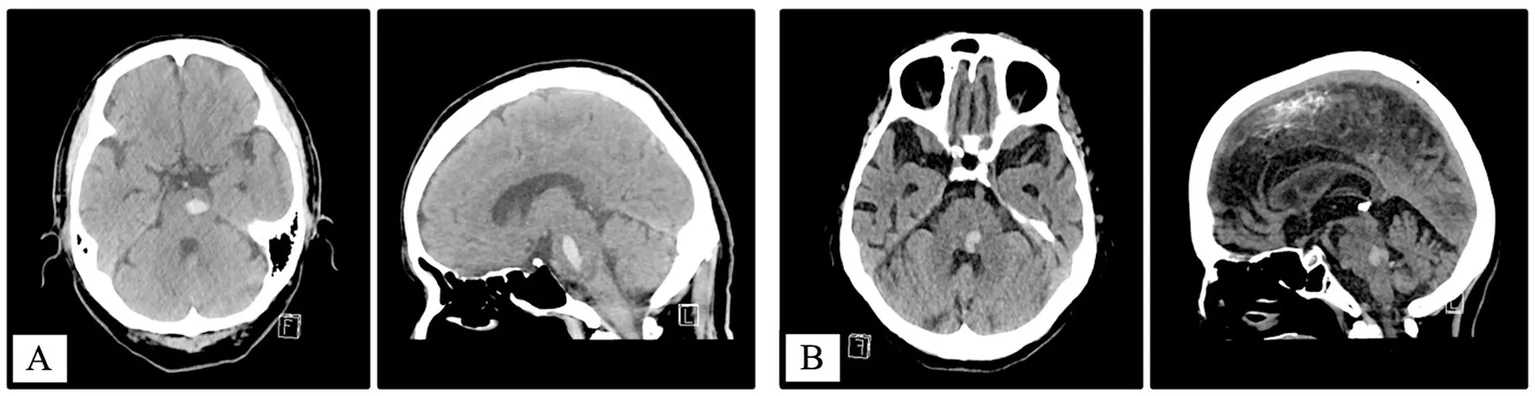
CT imaging of isolated infratentorial hemorrhage in two patients with more severe symptoms. **(A)** Axial and sagittal CT images of a 65-year-old male patient (NIHSS score of 35) presenting in a comatose state, showing a brainstem hemorrhage within the basal pons with a volume of 1 mL. **(B)** Axial and sagittal CT images of an 88-year-old female patient (NIHSS score of 8), demonstrating a brainstem hemorrhage located in the pontine tegmentum with a volume of 0.5 mL. Both cases highlight the small volume and severe clinical presentation of isolated infratentorial hemorrhage. NIHSS, National Institutes of Health Stroke Scale.

In patients with isolated infratentorial hemorrhage, 7 of 24 (29%) presented with NIHSS ≥7 and 16 (67%) had NIHSS <7 (one missing) (Supplementary material 4). Median hemorrhage volumes were similar between severity groups (5 mL in both NIHSS <7 and ≥7; Wilcoxon *p* = 0.80; Cliff’s *δ* = −0.076). Brainstem hemorrhages accounted for 5 of 7 severe presentations (NIHSS ≥7), whereas cerebellar hemorrhages predominated among mild presentations (11 of 15 NIHSS <7) (Figure 4).

Among patients with isolated infratentorial ICH, 6 of 24 patients met DTA-eligibility criteria (NIHSS ≥7 and time onset-arrival ≤360 min). Hemorrhage volume ranged from 1 to 26 mL with a median of 5 mL. In the exploratory slice-visibility analysis, hemorrhage was visible across a median of 7.5 (range, 4–11) axial slices on standardized CT reconstructions.

## 4 Discussion

In this large, single-center cohort of patients with spontaneous intracranial hemorrhage presenting with acute stroke symptoms, we found that hemorrhage size, location, and secondary extension were associated with NIHSS at admission. Although larger volumes were associated with higher NIHSS overall, this relationship varied substantially by location. Severe NIHSS scores were generally associated with larger volumes in lobar hemorrhages, but could occur at small volumes in infratentorial hemorrhage, particularly when the brainstem was involved. These findings provide epidemiological context for DTA workflows, in which reliable exclusion of intracranial hemorrhage before intravenous thrombolysis is essential and posterior fossa image quality on FDCT remains a potential concern.

Patients with NIHSS <7 had markedly smaller hemorrhage volumes than those with NIHSS ≥7 (6 mL vs. 38 mL, *p* < 0.001), consistent with prior studies linking larger hemorrhage volumes to greater clinical severity and higher NIHSS scores (23–25). However, volume alone did not fully capture clinical severity. Prior studies suggest that clinical impact of hemorrhage volume varies by location, with deep and infratentorial hemorrhages often producing greater neurological deficits than lobar hemorrhages at comparable volume (23, 26). Our findings align with this location-dependent relationship, as hemorrhage volume was strongly associated with NIHSS overall, but this association was less pronounced in infratentorial hemorrhage, where stroke severity appeared to depend more on location than volume alone. Cerebellar hemorrhages predominated in the NIHSS <7 group, whereas brainstem hemorrhages were more frequent in the NIHSS ≥7 group, potentially explaining the limited volume-severity relationship in this subgroup. Notably, infratentorial location was not associated with larger hemorrhage volume after adjustment (ß = −0.14, *p* = 0.52), indicating that location influences severity independently of volume rather than through larger bleeds. Small brainstem hemorrhages can produce severe deficits because of the eloquence of the affected area, not their size.

From a DTA workflow perspective, the most challenging subgroup comprises patients with symptom onset-to-arrival time ≤360 min, NIHSS ≥7, and isolated infratentorial hemorrhage. While FDCT achieves diagnostic accuracy comparable to MDCT for detecting intracranial hemorrhage, particularly in supratentorial regions (20–22, 27, 28), diagnostic confidence in the posterior fossa remains lower due to beam-hardening and motion artifacts (21, 22, 27–31). Although this subgroup was small in our cohort, it remains clinically important, as intracranial hemorrhage is a contraindication to intravenous thrombolysis and misclassification carries direct therapeutic consequences. The exploratory slice-visibility analysis showed that these lesions were visible across a median of 7.5 (range 4–11) weighted axial slices on standardized MDCT reconstructions, suggesting these lesions are generally not confined to a single-slice finding. However, multi-slice extent on MDCT reconstruction does not resolve the FDCT question, as posterior fossa image quality may be independently degraded by beam-hardening regardless of hemorrhage size. While the absolute frequency may limit its operational impact, the potential consequences of administering thrombolysis to a patient with undetected hemorrhage mean this risk cannot be dismissed and should be considered in DTA planning. Prospective paired MDCT-FDCT data are needed to determine actual detectability in DTA workflows.

Beyond ICH, the SAH subgroup showed a distinct pattern of clinical presentation. In a *post hoc* exploratory analysis, clinical severity at presentation among SAH patients differed markedly by etiology: all severely impaired patients had aneurysmal SAH, whereas non-aneurysmal cases had a median GCS of 15. This is consistent with the higher rate of loss of consciousness reported for aneurysmal SAH (32, 33), and with NIHSS being designed to capture focal deficits rather than the impaired consciousness or meningeal symptoms characteristic of SAH.

Our study has limitations. The retrospective design introduces the possibility of selection bias. The observed frequencies of hemorrhage type, location, and DTA-relevant subgroups may not generalize to drip-and-ship workflows, lower-volume centers, or regions with different stroke epidemiology. NIHSS was missing in 25 patients (6%), including 6 of 30 patients with SAH. Although the overall amount of missing NIHSS data was small, missingness was likely not completely at random because many patients without NIHSS had reduced consciousness based on available GCS scores. Exclusion of these patients may therefore have underestimated the proportion of severe presentations and attenuated associations between NIHSS and hemorrhage volume. Pre-morbid mRS was missing in 56 patients (14.2%) and was therefore excluded from primary analysis. Although pre-morbid mRS was only weakly associated with baseline NIHSS, and its exclusion may have introduced residual confounding, though the small effect size limits its likely impact. All analyses were exploratory without correction for multiple comparisons and should be considered hypothesis-generating. The small number of isolated infratentorial ICH (*n* = 24) limited the statistical power of subgroup analyses. Etiology classification in the non-aneurysmal SAH subgroup carries uncertainty. All 16 aneurysmal cases were confirmed by CTA, with 7 additionally undergoing DSA as part of treatment planning. In contrast, only 1 of the 8 unknown-etiology cases underwent DSA. A small underlying aneurysm, particularly involving a perforating vessel not reliably visualized on CTA, therefore cannot be excluded in the remainder. Any such misclassification would be expected to attenuate rather than exaggerate the observed association between aneurysmal status and clinical severity.

Furthermore, the slice-visibility analysis was exploratory and based on standardized MDCT reconstructions rather than paired MDCT and FDCT acquisitions. Therefore, our results cannot infer the sensitivity for infratentorial hemorrhage detection in FDCT. Nonetheless, our work has notable strengths, including a prospectively maintained stroke registry with near-complete regional catchment, standardized imaging protocols, semi-automated volumetric measurements to enhance reproducibility, and pre-specified analysis levels with DAG-guided covariate selection.

Prospective studies directly comparing MDCT and FDCT, such as the ongoing SPINNERS trial (34) (NCT05458908), are needed to validate these observations. Technical advances, including improved FDCT acquisition trajectories through sinusoidal C-arm movement, have improved infratentorial image quality (35), yet residual limitations in spatial resolution and contrast persist, warranting caution in high-stakes workflows such as DTA. Furthermore, advances in AI-based image enhancement and automated hemorrhage detection may help mitigate potential FDCT limitations, particularly in the posterior fossa. Beyond imaging considerations, adoption of DTA workflows is also influenced by operational factors such as angiography suite availability, staffing, and variability in pre-transfer imaging protocols.

## 5 Conclusion

Hemorrhage volume was strongly associated with clinical severity in ICH, but this relationship was location dependent. In isolated infratentorial hemorrhages, clinical severity appeared more closely related to anatomical location than to volume, with brainstem hemorrhages producing high NIHSS even at small volume. In direct-to-angiography strategies, the predominance of large-volume supratentorial hemorrhages among NIHSS ≥7 patients suggests that FDCT’s known posterior fossa limitations may be relevant to only a small minority of eligible cases. However, because detectability was not directly measured in this study, this remains a research gap. Prospective studies directly comparing FDCT and MDCT for posterior fossa hemorrhage detection are needed to confirm how often clinically relevant infratentorial hemorrhages would be missed in DTA workflows.

## Data Availability

The raw data supporting the conclusions of this article will be made available by the authors, without undue reservation.
