# Supplementary material for: Size and location of intracranial hemorrhage in acute stroke presentations: correlation with baseline NIHSS and relevance to direct-to-angiography workflows
Source: Front Neurol. 2026 Sep 8;17:1712607. doi: 10.3389/fneur.2026.1712607 (PMC13593524; doi:10.3389/fneur.2026.1712607)
Supplement: Supplementary file 1 [file Data_Sheet_1.PDF]

Supplementary Material

Supplement 1. CTA Protocol and Exploratory Slice-Visibility Analysis

CTA Protocol

CTA of the intracranial and cervical vessels was performed following intravenous administration of 40 mL iodinated contrast medium at a flow rate of 4 mL/s, followed by saline flush, with bolus tracking in the aortic arch. DSA was performed at the discretion of the treating interventional neuroradiologist when CTA findings were inconclusive or endovascular treatment was indicated. All images were permanently anonymised prior to analysis.

Exploratory Slice-Visibility Analysis

To contextualise the potential detectability of posterior fossa hemorrhages in DTA-eligible presentations, an exploratory slice-visibility analysis was performed in patients with isolated infratentorial hemorrhage who met clinical DTA eligibility criteria, defined as symptom onset-to-arrival time ≤360 minutes and NIHSS ≥7. CT images were reconstructed along the bicommissural line using 5-mm slice thickness and 3-mm interval. Hemorrhage visibility was quantified as the weighted number of axial slices containing visible hemorrhage, using a slice-weighting approach analogous to the ABC/2 method[1,2]: slices containing more than 75% of the maximal hemorrhage area were counted as 1 slice, slices containing 25–75% as 0.5 slices, and slices containing less than 25% as 0 slices. This analysis was intended to estimate hemorrhage visibility across standardised axial reconstructions and was not designed to determine FDCT diagnostic sensitivity.

Supplement 2. Directed acyclic graph for covariate selection in adjusted analyses.

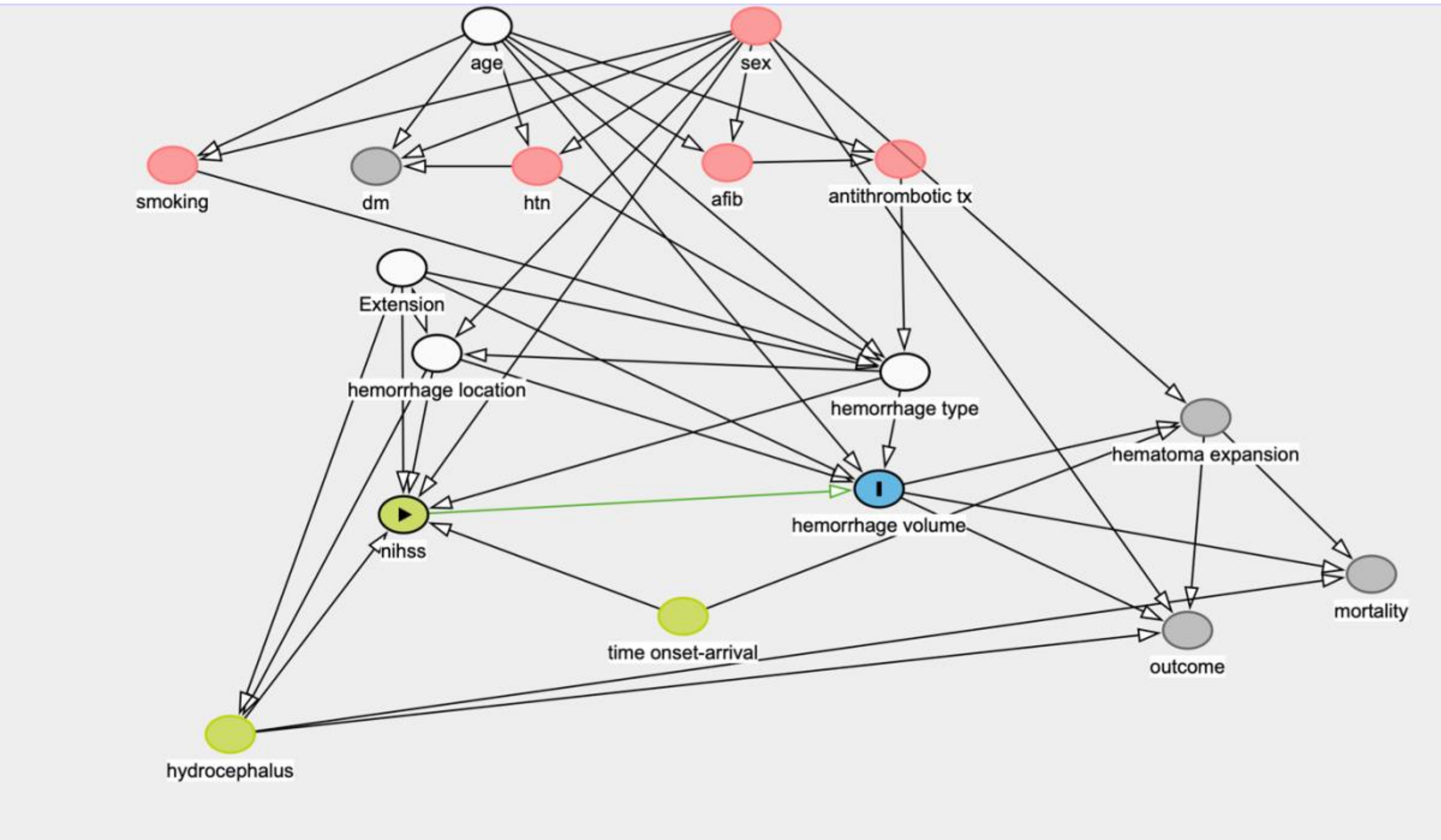

The DAG illustrates hypothesized relationships among demographic factors, vascular risk factors, hemorrhage characteristics, clinical severity (NIHSS), and outcomes. Based on this framework, the minimally sufficient adjustment set for analyzing the association between hemorrhage volume and NIHSS included age, hemorrhage location, and secondary extension.

htn = hypertension, dm = diabetes mellitus, extension = secondary extension of intracranial hemorrhage into other intracranial compartments, antithrombotic tx = antithrombotic treatment, afib = atrial fibrillation, smoking = active smoking defined as current use or within the past 2 years.

Supplement 3. Detailed Patient and Clinical Characteristics in IPH.

	Total		Supratentorial				Infratentorial			
			Lobar		Deep		Brainstem		Cerebellum	
**Count**	351		161		140		11		28	
Age	76	(63, 84)	76	(64, 82)	77	(63, 84)	65	(45, 93)*	74	(54, 93)*
Female sex	170	(48 %)	79	(49 %)	66	(47 %)	4	(36 %)	15	(54 %)
Premorbid mRS	1	(0, 2)	1	(0, 2)	1	(0, 1)	0	(0, 3)*	1	(0, 5)*
**Medical history**										
Ischemic stroke	61	(17 %)	30	(19 %)	23	(16 %)	1	(9 %)	3	(11 %)
ICH	32	(9 %)	17	(11 %)	12	(9 %)	1	(9 %)	1	(4 %)
Hypertension	276	(79 %)	118	(73 %)	117	(84 %)	6	(55 %)	25	(89 %)
Diabetes	64	(18 %)	27	(17 %)	26	(19 %)	2	(18 %)	5	(18 %)
Hypercholesterolemia	112	(32 %)	56	(35 %)	43	(31 %)	2	(18 %)	7	(25 %)
Smoking	56	(16 %)	27	(17 %)	20	(14 %)	0		5	(18 %)
Atrial fibrillation	78	(22 %)	32	(20 %)	31	(22 %)	2	(18 %)	11	(39 %)
Anticoagulation use	82	(23 %)	33	(20 %)	40	(29 %)	1	(9 %)	7	(25 %)
Antiplatelet use	92	(26 %)	44	(27 %)	30	(21 %)	3	(27 %)	9	(32 %)
**Clinical presentation**										
NIHSS	11	(4, 19)	12	(4, 19)	12	(5, 18)	11	(0, 32)*	3	(0, 32)*
GCS	14	(10, 15)	14	(10, 15)	14	(11, 15)	10	(3, 15)*	14	(3, 15)*
Wake up stroke	37	(11 %)	19	(12 %)	16	(11 %)	0		2	(7 %)
Transfer from referring hospital	25	(7 %)	12	(7 %)	7	(5 %)	1	(9 %)	3	(11 %)
Time (onset-admission)	159	(66, 553)	194	(65, 660)	136	(62, 407)	78	(25, 1789)*	329	(36, 7122)*
Systolic blood pressure	161	(140, 185)	158	(140, 180)	167	(148, 190)	160	(127, 216)*	176	(95, 220)*
**Outcome**										
In-hospital death	62	(18 %)	32	(20 %)	18	(13 %)	3	(27 %)	7	(25 %)
3-month mRS 0 – 2	97	(28 %)	38	(24 %)	41	(29 %)	4	(36 %)	12	(43 %)
3-month mRS 3 – 5	106	(30 %)	44	(27 %)	50	(36 %)	3	(27 %)	8	(29 %)
3-month mRS 6	122	(35 %)	63	(39 %)	43	(31 %)	3	(27 %)	8	(29 %)

ICH = intracranial hemorrhage, IPH = intraparenchymal hemorrhage, NIHSS = National Institutes of Health Stroke Scale, GCS = Glasgow coma scale, 3-month mRS = modified Rankin Scale score at 3-month follow-up, mRS score of 0 – 2 represents a good clinical outcome, 3 – 5 major disabilities, and 6 means death.

Quantitative data are presented as median (IQR) for variables with ≥30 observations; for variables with fewer than 30 observations (marked with *), the full range is given.

Supplement 4. Characteristics of Patients Imaged with Flat-Detector CT.

**Count**	10	
**Clinical data**		
Age	76.5	(52, 90)*
Gender, female	1	(10 %)
Premorbid mRS	0.5	(0, 2)*
GCS	12.5	(9, 15)*
NIHSS	20.5	(8, 23)*
**Hemorrhage type**		
Intraparenchymal	10	(100 %)
**Volume**		
Primary hemorrhage	14.5	(3, 50)*
Secondary extension	1.9	(0, 44)*
Total hemorrhage volume	14.5	(3, 88)*
**Hemorrhage location**		
Lobar	2	(20 %)
Deep	8	(80 %)
Infratentorial	0	
**Secondary extension**		
None	4	(40 %)
Intraventricular	6	(60 %)
**Mass effect**		
Midline shift (n, %)	5	(50 %)
Midline shift (in mm)	1.5	(0, 10)*
Herniation	1	(10 %)
Hydrocephalus	4	(40 %)

Quantitative data are presented as median (IQR) for variables with ≥30 observations; for variables with fewer than 30 observations (marked with *), the full range is given.

Supplement 5. Bleeding Characteristics and Anatomical Location in Intraparenchymal Hemorrhage.

	Total		Intraparenchymal hemorrhage					
			Lobar		Deep		Infratentorial	
**Count**	351		161		140		11	
**Volume**								
Primary hemorrhage	16	(5, 44.5)	30	(8, 66)	9	(3, 22.2)	2	(0.3, 19.1)*
Secondary extension	0.5	(0, 7.3)	0.5	(0, 6)	0	(0, 7.1)	0	(0, 18.3)*
Total volume	20	(5, 54)	38	(12, 78)	11.6	(4, 41.2)	6	(0.3, 37.4)*
**Hemorrhage level**								
Isolated supratentorial	251	(72 %)	137	(85 %)	107	(76 %)	0	
Isolated infratentorial	23	(7 %)	0		2	(1 %)	7	(64 %)
Supra- and infratentorial	77	(22 %)	24	(15 %)	31	(22 %)	4	(36 %)
**Location of secondary hemorrhage extension**								
Intraparenchymal	20	(6 %)	12	(7 %)	5	(4 %)	0	
Subarachnoid	95	(27 %)	71	(44 %)	12	(9 %)	1	(9 %)
Intraventricular	147	(42 %)	59	(37 %)	59	(42 %)	6	(55 %)
Subdural	30	(9 %)	25	(16 %)	2	(1 %)	0	
**Mass effect**								
Midline shift (n, %)	143	(41 %)	83	(52 %)	51	(36 %)	0	
Midline shift (in mm)	0	(0, 6)	3	(0, 8)	0	(0, 5)	0	(0, 0)*
Herniation	105	(30 %)	64	(40 %)	25	(18 %)	1	(9 %)
Hydrocephalus	94	(27 %)	44	(27 %)	35	(25 %)	2	(18 %)

Quantitative data are presented as median (IQR) for variables with ≥ 30 observations; for variables with fewer than 30 observations (marked with *), the full range is given.

Supplement 6. Location-stratified Spearman rank correlation between primary hemorrhage volume and admission NIHSS (Tier 2, n = 333)

Location	n	Spearman ρ	95% CI†	p-value‡
Deep	139	0.675	0.566–0.769	<0.001
Lobar	156	0.658	0.547–0.747	<0.001
Infratentorial	38	0.323	0.041–0.576	0.048

† Bootstrap 95% percentile CI (R = 2000 resamples). ‡ From Spearman's test (cor.test, two-sided); infratentorial p-value approximate based on t-approximation. Primary analysis restricted to IPH patients with complete NIHSS and vol_prim > 0. Six patients with unclassified location excluded from stratified analysis.

Supplement 7. Primary (Isolated Infratentorial) Intraparenchymal Hemorrhages Stratified by NIHSS <7 and ≥7.

	All IPH		Isolated Infratentorial IPH	
	NIHSS < 7	NIHSS ≥ 7	NIHSS < 7	NIHSS ≥ 7
Count	116	217	16	7
Age	73 (60, 81)	77 (66, 84)	67.5 (45, 93)*	65 (53, 88)*
Female sex	58 (50 %)	104 (48 %)	7 (44 %)	3 (43 %)
Premorbid mRS	1 (0, 1)	1 (0, 2)	1 (0, 3)*	1 (0, 3)*
**Clinical presentation**				
NIHSS	3 (1, 4)	17 (12, 24)	2 (0, 5)*	32 (7, 32)*
GCS	15 (14, 15)	12 (10, 14)	15 (12, 15)*	4 (3, 14)*
Transferring from referring hospital	25 (7 %)	14 (12 %)	322 (17, 3907)*	66 (0, 809)*
Time (onset – admission)	117 (58, 346)	245 (73, 648)	152 (95, 200)*	147 (129, 220)*
**Hemorrhage Type**				
Parenchymal	5 (2, 13)	28 (9, 60)	15 (94 %)	7 (100 %)
Subarachnoid	0 (0, 0)	1 (0, 1)	1 (6 %)	0
Subdural	55 (38 %)	128 (51 %)	NA	NA
Intraventricular	43 (29 %)	111 (44 %)	NA	NA
**Hemorrhage Volume**				

Primary IPH	5 (1.4, 12.5)	26 (9, 60)	5 (0.1, 15)*	5 (0.5, 26)*
Secondary IPH	0 (0, 0)	2 (0, 9)	0 (0, 2)*	0 (0, 2)*
Total Volume	5 (2, 14.3)	37.4 (12, 78)	5 (0.1, 15)*	6 (0.5, 28)*
**Location of primary ICH**				
Lobar	51 (44 %)	105 (48 %)	NA	NA
Deep	42 (36 %)	90 (41 %)	NA	NA
Infratentorial	23 (20 %)	15 (7 %)	16 (100 %)	7 (100 %)
**Location of secondary ICH**				
Intraparenchymal	85 (73 %)	76 (35 %)	15 (94 %)	4 (57 %)
Subarachnoid	14 (12 %)	31 (14 %)	0	2 (29 %)
Intraventricular	11 (9 %)	70 (32 %)	0	1 (14 %)
Subdural	6 (5 %)	40 (18 %)	1 (6 %)	0
**Space-occupying effect**				
Midline shift, count	9 (8 %)	121 (56 %)	0	0
Midline shift, median	0 (0, 0)	3 (0, 8)	NA	NA
Herniation	7 (6 %)	86 (40 %)	0	1 (14 %)
Hydrocephalus	8 (7 %)	75 (35 %)	0	1 (14 %)
**Outcome**				
In-hospital death	2 (2 %)	52 (24 %)	0	2 (29 %)
3-month mRS				
mRS 0-2	70 (60 %)	26 (12 %)	12 (67 %)	1 (14 %)
mRS 3-5	30 (26 %)	73 (34 %)	4 (22 %)	2 (29 %)
mRS 6	11 (9 %)	100 (46 %)	1 (6 %)	3 (43 %)

3-month mRS of 0 - 2 represents a good clinical outcome, 3 - 5 major disabilities, and 6 means death.

Quantitative data is presented as median (IQR) for variables with ≥ 30 observations; for variables with fewer than 30 observations (marked with *), the full range is given.

Supplement 8. Mean intracerebral hemorrhage volume by NIHSS score intervals.

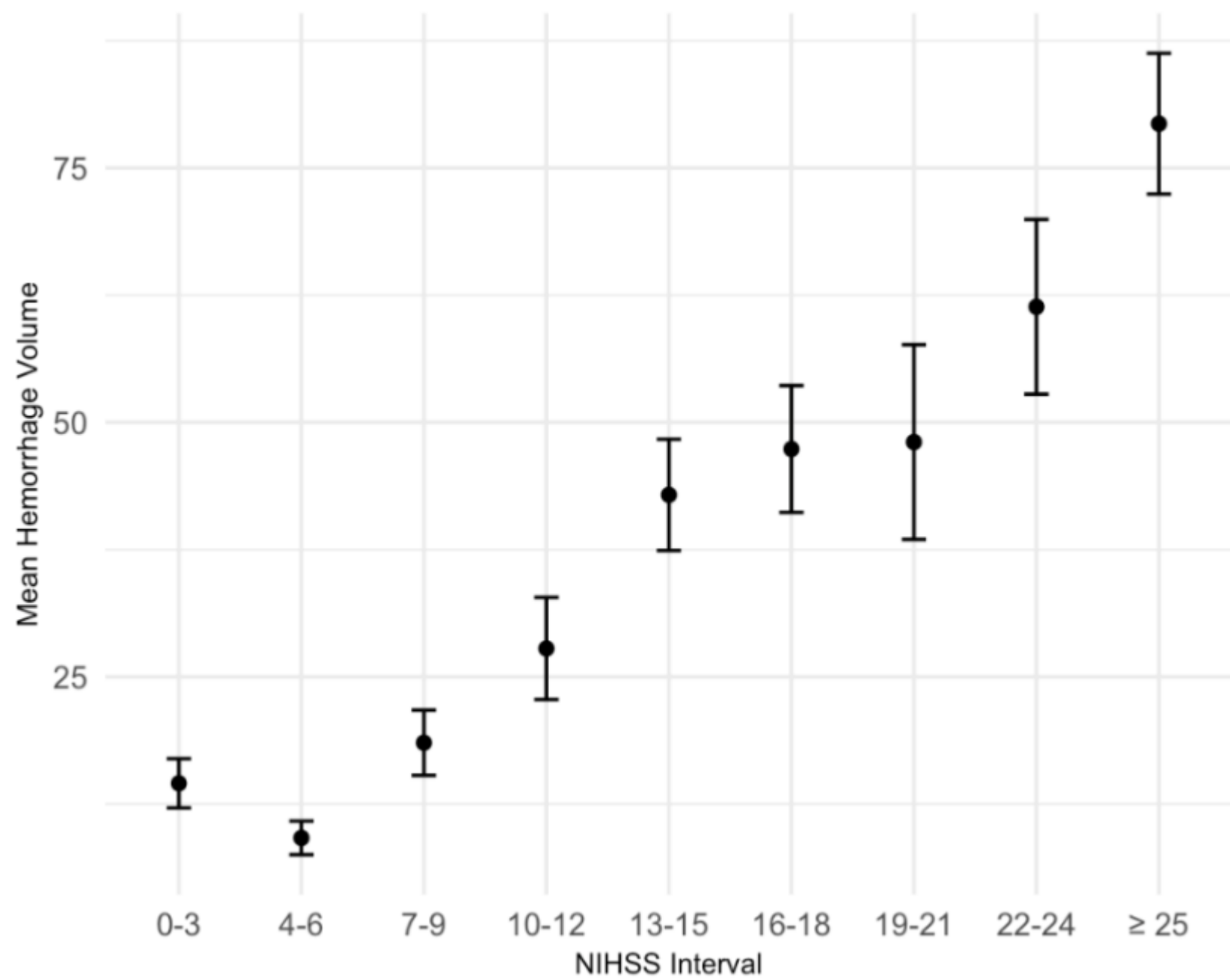

Each point represents the average hemorrhage volume (ml) observed within the specified NIHSS range, and vertical bars denote the 95% confidence interval around the mean. Hemorrhage volumes increase progressively with higher NIHSS scores, indicating a positive association between stroke severity and hemorrhage volume.

NIHSS = National Institut of Health Stroke Scale.

References

1. Huttner HB, Steiner T, Hartmann M, Köhrmann M, Juettler E, Mueller S, Wikner J, Meyding-Lamade U, Schramm P, Schwab S, et al. Comparison of ABC/2 Estimation Technique to Computer-Assisted Planimetric Analysis in Warfarin-Related Intracerebral Parenchymal Hemorrhage. Stroke. 2006;37:404-408. doi:10.1161/01.STR.0000198806.67472.5c
2. Kothari RU, Brott T, Broderick JP, Barsan WG, Sauerbeck LR, Zuccarello M, Khoury J. The ABCs of Measuring Intracerebral Hemorrhage Volumes. Stroke. 1996;27:1304-1305. doi:10.1161/01.STR.27.8.13
